# Breaking Boundaries: Interdisciplinary Workshops to Write Integrated Medical School Assessments

**DOI:** 10.1007/s40670-022-01647-1

**Published:** 2022-10-03

**Authors:** Maresa Harvey, Petra Dimmers, Mohammed Ahmed Rashid

**Affiliations:** UCL Medical School, GF/664, Royal Free Hospital, London, NW3 2PR UK

## Abstract

Although integrated medical school curricula have been widely adopted and examined, there has been comparatively less attention on the challenges associated with developing integrated assessments. Working with medical schools around the world, we have developed a writing workshop format that unites teachers from different disciplines to produce integrated assessment items.

University College London Medical School (UCLMS) is engaged in a number of international collaboration projects designed to develop and improve medical education institutions and programmes around the world [1, 2]. A key focus of these projects is building local capacity, and as such, they include faculty development events either at the UCLMS campus in London, or on site at the local institutions of our collaborators, although restrictions on international travel caused by the COVID-19 pandemic have meant these have most recently taken place virtually.

One important strand of these projects is the provision of support in the areas of medical school assessment design and delivery, including providing assistance to develop defensible assessment policies and banks of high-quality written and practical assessment items. Indeed, faculty development in assessment has been conceptualised as the ‘missing link’ in driving forward competency-based medical education [3]. We have observed a pattern throughout our collaboration projects that when establishing a new medical school or transforming an existing one, the task of developing a high-quality assessment approach is one of the most demanding. Matching integrated curriculum design with an integrated assessment approach, weaving together different disciplinary areas in individual exams and even individual questions, is a particularly challenging feat.

Recognising that many outstanding medical teachers and experts can struggle with the demands of developing high-performing written and practical assessment items, and that the traditionally disciplinary nature of university and hospital departments can sometimes limit collaborative working, we have developed a faculty development workshop approach that prioritises team working. Following a focussed review of the theory and evidence for using single best answer knowledge tests and objective structured practical assessments, faculty teams are divided into groups of 3 or 4, with purposefully chosen teams to bring together a broad range of disciplinary expertise. After spending time generating assessments that integrate knowledge and skills from across their curricular and disciplinary areas, teams then present a selection of their items to the entire group for discussion and feedback. There are no specific targets about the number of items and the focus is on quality rather than quantity.

An example team might include three teachers all contributing to an integrated undergraduate medicine module: a paediatrician; an immunologist; and an anatomist. Whereas the paediatrician bring important skills in writing authentic clinical scenarios to reflect ‘real’ practice, the immunologist brings an in-depth understanding of the key scientific mechanisms and concepts that need to be tested through knowledge tests, and the anatomist brings expertise in the types of hands-on learning that students will have encountered and thus can be tested in a practical assessment station. All three contributors bring important perspectives about the appropriate standard for the year group. By probing, advising, and challenging one another, they are likely to produce more innovative and intersecting assessments than had they worked individually.

Not only does team working make a demanding activity more engaging, it also reinforces interdisciplinary bridges, strengthens relationships across teams, encourages ‘out of the box’ and creative thinking, and ultimately helps to produce assessments that authentically integrate different curricular areas.

In person, these workshops work well in large classrooms, with teams clustered around tables to promote collaborative working followed by group feedback. Since early 2020, we have adapted these workshops using virtual platforms such as Microsoft Teams (using the ‘breakout room’ function), Microsoft Forms (enabling teams to share completed assessment items with facilitators in real time for feedback and discussion), and Miro (allowing teams to complete ‘flipchart’ style brainstorming exercises). Without travel constraints, these workshops have been able to go ahead with less lead-in time than usual, allowing them to be more responsive to rapidly changing needs of local teams. Our usual approach of running visits for typically 2 to 4 full days consecutively has been adapted to a more flexible approach that can span several weeks. This more distributed timetable has given participants more time between sessions to consolidate their development, practise writing items individually, and bring back troublesome items for further feedback and assistance. Although technical aspects of training have been possible through virtual platforms, both facilitators and participants have noted that the ‘softer’ benefits of informal advice and support offered in individual, unscheduled conversations, have been sorely missed.

Assessment writing workshops have the potential to be tedious and laborious at the best of times, and perhaps more than ever when delivered online. By focussing on maximising interactivity and inter-disciplinary teamwork, though, we have managed to develop a workshop format that has not only been popular with participating faculty teams, but has also been highly productive in generating high-quality assessment items suitable for use in summative medical school exams.
